# Exploring the usability and perceived benefits of Brain Health PRO: An online educational program for healthy brain aging

**DOI:** 10.1177/20552076251395585

**Published:** 2025-11-19

**Authors:** George Philip, Alexandra J Fiocco, Sylvie Belleville, Howard Chertkow, Howard H Feldman, Manuel Montero-Odasso, Haakon B Nygaard, Marie Y Savundranayagam, for the Canadian Consortium on Neurodegeneration in Aging (CCNA), CAN-THUMBS UP Study Group

**Affiliations:** 1 School of Health Studies, 6221 Western University, London, ON, Canada; 2 Sam Katz Community Health and Aging Research Unit, 6221 Western University, London, ON, Canada; 3 Department of Psychology, 7984 Toronto Metropolitan University, Toronto, ON, Canada; 4 Centre de recherche de l’Institut universitaire de gériatrie de Montréal, Montréal, QC, Canada; 5 Département de psychologie, 5622 Université de Montréal, Montréal, QC, Canada; 6 113635 Lady Davis Institute for Medical Research, Montréal, QC, Canada; 7 Department of Medicine (Neurology), 7938 University of Toronto, Toronto, ON, Canada; 8 63670 Rotman Research Institute, Baycrest Academy for Research and Education, Toronto, ON, Canada; 9 Department of Neurology and Neurosurgery, 5620 McGill University, Montréal, QC, Canada; 10 Department of Neurosciences, 21814 University of California San Diego, San Diego, CA, USA; 11 Alzheimer's Disease Cooperative Study, School of Medicine, University of California, San Diego, CA, USA; 12 151158 Lawson Health Research Institute, London, ON, Canada; 13 Gait and Brain Lab, 60446 Parkwood Institute, Western University, London, ON, Canada; 14 Departments of Medicine and Epidemiology and Biostatistics, 6221 Western University, London, ON, Canada; 15 Department of Medicine, Division of Neurology, 8166 University of British Columbia, Vancouver, BC, Canada

**Keywords:** Brain health, dementia, prevention, online intervention, digital health

## Abstract

**Background:**

Brain Health PRO (BHPro) is a 45-week online educational program designed to promote healthy lifestyle behaviors and reduce dementia risk. Available in both English and French, it provides information and guidance on seven modifiable dementia risk factors: physical activity, cognitive engagement, nutrition, sleep, social and psychological health, vascular health, and vision and hearing. Upon completing a short intake questionnaire, users receive a personalized risk profile to guide their priorities and goals, with ongoing feedback throughout the program.

**Objective:**

This study aimed to explore the usability and perceived benefits of BHPro over a 12-week period.

**Methods:**

Eight community-dwelling older adults across Canada (ages 68–83; four male, four female) pilot-tested the program. Virtual focus groups were conducted at two time points: after 6 weeks and after 12 weeks of participation. Transcripts were analyzed using thematic analysis, organizing codes into sub-themes and themes.

**Results:**

Three main themes emerged: (1) Content—participants appreciated the likability, accessibility, and informativeness of the program; (2) Mechanics—comfort with technology, the usefulness of program features, and enjoyment of narration and graphics; (3) Engagement and Learning—participants reported increased motivation, new knowledge, behavioral changes, and desire for continuous feedback.

**Conclusion:**

Participants shared positive experiences with the online content and interface, benefitting on new knowledge gained while highlighting behavioral changes. The need for greater personalization in future iterations was emphasized. The BHPro pilot was well received by older adults, who found the platform usable, engaging, and beneficial. Findings offer insights into using online platforms to support brain health.

## Introduction

It is estimated that over 500,000 individuals in Canada live with neurodegenerative dementias, with projections indicating a doubling of this figure by the year 2031.^1,2^ Currently, there are limited treatment options for the various neurodegenerative dementias.^3^ Although advancing age presents as the most prominent non-modifiable risk factor for developing dementia,^2,4^ nearly 40% of the population attributable risk of dementia is associated with modifiable risk factors, including social isolation, physical activity, sleep, sensory impairments, dietary habits, as well as metabolic and vascular risk profiles.^5^

The incorporation of digital tools and technology into health prevention and education has shown to significantly improve accessibility and scalability.^6–8^ Previous research indicates that even during the early stages of Alzheimer's disease (AD), older adults can gain significant benefits from formal educational programs related to dementia.^7^ Such courses have been shown to enhance understanding of the disease, positively impact their mood, self-esteem, and increase their sense of self-efficacy.^7^ Additionally, web-based educational initiatives addressing risk factors and protective measures have demonstrated efficacy in improving dementia-related risk profiles among middle-aged adults.^9^ Participation in educational programs can potentially boost dementia literacy, enhance personal empowerment, and increase general self-efficacy, all of which contribute to a greater engagement in strategies for promoting brain health.^9,10^ These effects suggest that educational interventions might not only reduce dementia risk directly but also improve participants’ readiness to adopt preventative measures and increase their motivation to engage in ongoing dementia prevention research. In addition, a modest delay of 1 year in the onset of dementia has been projected to save the Canadian health care system $120 billion over the next three decades.^11^

### Brain health support program for dementia prevention

There is a critical gap and need for dementia education given Canada's rapidly growing aging population.^12^ Recognizing the potential of technology to influence dementia prevention, there are currently no large-scale online or digital dementia prevention programs available in Canada. To address this, the Canadian Therapeutic Platform Trial for Multidomain Interventions to Prevent Dementia (CAN-THUMBS UP) developed an interactive Brain Health Program (BHPro), a digital, online educational intervention designed to promote healthy lifestyle behaviors to reduce risk of and/or prevent dementia.^13^ The current qualitative study explores the usability and perceived benefits of BHPro from participants who took part in a 12-week pilot study. This pilot was part of a broader effort to iteratively improve the program before its use in a larger study.

## Methods

### Study design

A mixed-methods pilot study was conducted to assess usability and acceptance of the *Brain Health PRO/Santé Cerveau PRO* over a 3-month period. A total of 20 participants across Canada pilot tested BHPro over a 12-week period between November 2021 and April 2022. During the pilot, participants received four chapters per week, resulting in 48 chapters over 12 weeks, simulating the structure of the full program, which is designed to span 11 months. Each chapter requires approximately 20–30 minutes to complete, corresponding to a weekly time commitment of approximately 1.5–2 hours and a total expected commitment of 24–36 hours across the study period. Although no formal quantitative measure of burden was collected, participants were invited to share their experiences and reflections on workload during focus groups. In addition to exploring program engagement, participants completed questionnaires and provided feedback on their experience. This feedback was integral to personalizing the program and prioritizing content to align with user needs.

The program covers eight content topics:^10,13^ (1) general information on cognition, dementia, genetic risk, and healthy lifestyle; (2) physical activity; (3) cognitive engagement; (4) nutrition; (5) sleep; (6) social and psychological health; (7) vascular health; (8) vision and hearing.

From the pilot sample, eight participants were invited to participate in two focus group sessions, one at 6 weeks (halfway point) and another at 12 weeks (end of program). This study was approved by the research ethics boards at the University of British Columbia (#H20-02817), University of Victoria (#BC20-0580), Clinical Trials Ontario (#3497), and the Horizon Health Network (#2022-3096).

This paper reports the qualitative component of the project, which was designed to explore the usability and perceived benefits of BHPro. A quantitative study assessed user engagement and outcomes, with a description of the program's design process to develop BHPro.^14^ Together, these components provide a holistic evaluation of BHPro, integrating qualitative insights with quantitative findings to inform its overall effectiveness and implementation.

### Recruitment and selection of participants

Participants interested in enrolling in the BHPro Pilot had to meet specific criteria to ensure their eligibility and suitability for the study. Inclusion criteria entailed: (1) ages 60–85 years; (2) meeting cognitive criteria as defined by the Canadian Consortium on Neurodegeneration in Aging (CCNA), falling into one of the following categories: Cognitively Intact (CI), cognitively intact with Subjective Cognitive Impairment (SCI), or Mild Cognitive Impairment (MCI); (3) be classified as being at increased risk of dementia based on specific risk factors, namely having a first-degree family history of dementia or documented midlife risk factors, including hypertension, hypercholesterolemia, high body mass index (BMI), or physical inactivity; (4) proficiency in English to engage effectively with the program's content and materials; (5) possession of technical skills required to navigate an online platform, including access to a computer with internet connectivity, proficiency in email communication, and the capability to complete remote assessments; (6) adequate vision and hearing for interacting with the program's multimedia components; (7) ability to sit comfortably for approximately 30 minutes during instructional sessions. These criteria ensured that participants were well-suited for engaging with the program's educational content and contributed effectively to the study's objectives regarding dementia risk assessment and prevention strategies.

All 20 participants who completed the BHPro pilot program were invited to participate in two focus groups. 15 individuals consented and were available to participate. This cohort, however, was not evenly distributed, with a disproportionate number from Ontario, the majority being female, and primarily residing in urban settings. A purposive sampling approach was adopted to balance representation across provinces, rural and urban settings, sex, and years of education, thereby maximizing demographic and regional heterogeneity while preserving feasibility for robust qualitative inquiry. Eight participants were selected and provided written informed consent prior to study initiation. This is in line with qualitative research standards, guided by methodological recommendations that 5–8 participants per focus group are optimal for generating in-depth and interactive discussion.^15^

### Data collection

Two focus groups, each being 2 hours in duration, were conducted to explore various aspects of the program, with only the researcher and participants in attendance. No relationships were established prior to study commencement. The first focus group explored general experiences, such as program accessibility, likability, and overall feedback. The second focus group focused on the perceived benefits of BHPro, program personalization, barriers, future engagement, and participants’ potential role as BHPro experts.

The core discussion across both focus groups centered on program accessibility, usability, and perceived benefits, despite the different topic discussions. This was intentional as concentrating on multiple topics in a single focus group can likely overwhelm participants and limit the richness of dialogue. Participants were able to engage more deeply with each topic, which led to more detailed and insightful responses. This approach aligns with methodological recommendations that multiple sessions can enhance data richness and support data saturation while maintaining participant engagement.^16^

Interviews from the focus groups were transcribed verbatim and analyzed using thematic analysis. Transcripts were not returned to participants for feedback or correction. After each focus group, field notes were documented, and reflexivity was integrated across all stages of the study. Researcher positionality, experiential background, and potential biases were examined as part of this process. A codebook was first generated following an independent review of the transcripts. Codes were extracted from the transcripts and were organized into themes and sub-themes manually using a recursive process until data saturation was achieved. Relevant quotes were then extracted for each theme and sub-theme regarding participants’ experiences and perceptions of the BHPro program.

## Results

### Participant demographics

As shown in Table 1, the sample included four males and four females with a balanced educational background and geographic representation.

**Table 1.** Participant demographics.

Total participants	*N* = 8
Age range	68–83
Sex	Male (*n* = 4) Female (*n* = 4)
Education in years	12–18 years
Rural or urban	Rural (*n* = 5) Urban (*n* = 3)
Location	British Columbia (*n* = 3)
	New Brunswick (*n* = 3)
	Ontario (*n* = 2)

### Qualitative findings

Thematic analysis revealed three overarching themes: (1) Content, with subthemes of likability and accessibility, and the informative nature of the program; (2) Mechanics, with subthemes of technology comfort and importance of tech support, utility of program features, enjoyment of narration and graphics; and (3) Engagement and Learning, with subthemes of motivation, new knowledge, behavioral changes, perceived benefits and need for continuous feedback. The program was co-created with a Citizen Advisory Group (CAG)^17^ and developed with the support of a Knowledge Translation (KT) expert to ensure its accessibility and alignment with user needs. All extracted quotes by theme and subtheme are presented in Supplemental Table 1.

**Theme 1a**: Content—Likability and Accessibility. All participants found the content of BHPro to be likable and accessible. One participant shared:

I think you have an unlimited number of people who are looking for ways to curtail what they're observing within their own thinking processes decreasing and I think again the positivity of the program is wonderful and I think you would find huge buy-in from people who are looking for ways to improve the quality of their life and maintain their brain power. (P5)

Participants shared that their experience with BHPro was “wonderful,” “excellent,” “positive,” “promising,” and “helpful.” One participant stated, “I found [BHPro] incredibly useful and helpful, and I think it's going to be great for the community” (P7).

Participants found the content to be accessible. One participant shared, “I really particularly enjoy the mix of sort, of the medical information along with the recommendations” (P6), which were “simply stated” and “direct.” Participants also appreciated the number of examples used within in each topic because “that really helps you understand the context” (P1).

Although most participants found the language to be accessible, they reflected on possible challenges for other older adults who have a lower literacy level or English as a second language. One participant shared, “I live in a rural community with many people here who have not a very extensive education, the language would detract from their willingness or wanting to be part of the program” (P5). Another participant shared,

“There might be some questions with vocabulary in some cases that people are not sure what cognitive impairment means or you know those kinds of terms, even dementia you know maybe they need to be defined” (P2). One participant shared their difficulty with some of the “medical” terms used and suggested, “maybe a glossary of terms would be helpful” (P6). Together, this dialogue underscores the importance of clear communication in health programs.

**Theme 1b**. Content—Informative Nature of the Program. The detailed coverage of sleep, nutrition, social networks, and mental and physical health was particularly well-received. Participants appreciated the breadth of topics and the fact that the information shared was created by experts in the field. One participant shared:

I found the information good and as someone else mentioned, it wasn’t Google type information; it was relevant, it was supported, it was from articles that were peer reviewed, so yes I had a lot of respect for the creators of this program. (P1)

Participants appreciated the value of information and the recommendations provided for dementia risk reduction. One participant stated, “I found [BHPro] very educational, and I know that I’ve learned things that I’m going to continue to use…” (P5). Another participant shared:

It's very positive and it also gives you some solutions like, you know, doing the list of things, write them down, do things that help you remember, lower your cholesterol level, all of those things are important and they’re all doable, so I can’t speak highly enough of how well I think this would lend itself to community. (P1)

**Theme 2a**: Mechanics—Design*.* The design of BHPro, including its user interface, narration, and visual elements, was well-received. Participants noted the attractiveness and usability of the website, the quality of narration, and the effectiveness of visual graphics. With respect to quality of the graphics, one participant shared, “I think the graphics are great, the colour is fresh, it's uplifting, the photography is great, the font is easy to read. From a design standpoint, I find it very appealing” (P3). Speaking to the quality of the narrator, one participant shared, “I really felt he was excellent … very good and his allocution was good and I found it calming … but it was nice that he just had such a level cadence the whole time that it was very easy to follow him” (P5). Another participant said, “You couldn’t have had a better narrator” (P4). The ability to move back to previous chapters was also noted as a strength of the program design. As one participant shared: “you could always go back and have a look, which was a great set up on that program; that if anybody wanted review anything they could go back in and do it” (P3). Some participants also appreciated the text reminders to complete the weekly program topics. One participant explained “it was helpful the one day … I totally forgot about doing it, that was the one time, and I heard the noise a couple of times and I thought, oh I didn't do this” (P4).

In discussing some of the user-specific elements of the program interface, participants did not find the digital notebook useful as a way to track information related to their goals and objectives. One participant shared, “I didn’t find that column to put your notes in very useful either and didn’t use it at all in the end. It didn’t seem to me that it would enhance anything I was getting out of the program” (P2). Another participant shared, “I think as a user I would find it helpful for somebody to say, well what we've introduced this week is notebook and this is what you're supposed to do with it, as opposed to just finding it there” (P3). However, participants appreciated the personal lifestyle risk profile that is generated following risk assessment. One participant shared:

the speedometer showing you, you know red yellow or green sort of thing, was a quick synopsis, so this is where I'm at and where I have to work on, so I thought the personalization was actually there right from the start. (P7)

Some participants requested greater personalization of the program with respect to specific chapters that are offered to align with the individual's generated risk profile and specific recommendations tailored to the individual risk profile that considers their location (e.g., exercise during the winter months). One participant shared, “it would be useful to have a pin pointed to me to say more specifically, you know you're not as physically active as you should be and here are some suggestions” and “what foods am I missing, what sorts of activities should I be increasing” (P7). One participant concluded “…I think personally I feel that the personalization end of it could be improved to be of more value” (P1).

**Theme 2b**: Mechanics—Technical Issues. Despite the overall positive feedback, some technical challenges were reported, such as glitches in website navigation and device malfunctions. These issues highlight a gap in the program's usability, as technical problems can hinder user experience and engagement. These glitches were related to navigating the website, where clicking on icons were not working as swiftly or were freezing. For example, one participant shared, “I found some glitches that were technical in nature, and we worked through those and they were resolved, but they are the only barriers that I see,” and “I had to reload the website a couple of times. I uninstalled, reinstalled it. My password wouldn’t work and they assigned a new password and so on and so forth…” (P3).

Participants were grateful for the technical support, accessibility, and swift responsiveness to their technical issues: “I had the same sort of technical glitches, but they were always solved really quickly so the support was great” (P5). The quick resolution of technical problems by support staff was a notable positive aspect, emphasizing the importance of responsive customer service in addressing user concerns.

**Theme 3a**: Engagement and Learning—Motivation and Behavioral Changes.

Participants were interested in supporting their own health, and some had a parent or family member with dementia which motivated their engagement with the program. One participant shared, “I found it fully engaging… I certainly am more aware of what I need to do for the future, it helps set the path for where I'm going so I found it very helpful and I’m very glad to have participated…” (P7). Participants also saw the program as being proactive. One participant shared:

I find it very positive very proactive very much geared towards saying to people you can make a difference, you need to put the effort into it but the effort will be rewarded, and I think that's what we all need to hear that it is good. (P5)

BHPro encouraged participants to expand their social network, either through volunteering or connecting with family and friends. One participant shared, “I find when I'm out, I'm more chatty, and willing to engage other people. I’ve re-contacted some old chums and have got together with them so the engagement with that program did it for me” (P8). BHPro also provided a sense of acknowledgment and encouragement of participants’ existing heathy lifestyle efforts. For example, one participant shared:

I really look at how much sleep I really do get, so in the morning more so than I did before, so I know that I need that 6–8 hour sleep, and I usually do, I have always got that, but I make sure a little more. (P3)

**Theme 3b**. Engagement and Learning—Perceived Benefits & Continuous Feedback. Participants noticed personal benefits and began to share BHP with their family, friends, and relatives. One participant shared:

I found it interesting that I’ve shared a lot of the information with [spouse] who is 10 years older than I am and it's been kind of an interesting happy hour conversation quite frequently, just some of the changes that we could be making as a couple. (P5)

Finally, continuous engagement and the need for feedback on their brain health were discussed by participants. While improvements on person risk scores were provided by BHPro, participants were curious about improvements in their brain health after making behavioral changes to decrease their risk score. As one participant shared:

I would definitely favor continuing it and expanding it, and I think it was useful and I think the time frame for me was about right I wouldn't want it to continue to go for months but you know getting a little sample every once in a while, maybe twice a year or something, and then getting possibly feedback would be useful. (P2)

This feedback highlights the value of offering boosters or refreshers, rather than extending the program over an 11-month period, with periodic updates to reinforce learning and provide ongoing motivation.

## Discussion

As the demand for digital solutions in health and education grows, online interventions have emerged as powerful tools for promoting well-being among older adults.^18^ These programs offer the potential to reach and engage individuals who are increasingly spending more time at home, whether for learning, work, or leisure.^7^ The effectiveness of such interventions, such as BHPro, hinges not only on the quality of their content but also on how well they are designed and delivered. As the first large-scale online educational program of its kind that offers a wholistic approach to dementia risk reduction, the current study offers important insight into program implementation and key elements that contribute to effective programming. Exploring the usability, likability, and perceived benefits of BHPro provides valuable insights into optimizing online health programs, specifically those designed to promote brain health. Creating programs that produce meaningful health outcomes, such as reducing the risk of dementia, depends on their ability to engage users and promote sustained adherence and use.

### Content

The content of BHPro was positively received by all participants who engaged in the pilot testing of the program, largely due to its appeal, accessibility, and informative nature. Content is a critical component of any online educational tool as it profoundly impacts user engagement and the overall efficacy of the learning experience.^19^ Moreover, content must be accurate, comprehensive, up-to-date, and relevant to users.^19^ The integration of these elements ensures that learners acquire practical knowledge applicable to their everyday lives, thereby enhancing the digital tool's practical utility.

BHPro was specifically designed to promote healthy brain aging and mitigate the risk of dementia among older adults. Consequently, the program is particularly relevant to older adults at risk for cognitive impairment and individuals with a family history of cognitive impairment. BHPro addressed this by focusing on key areas such as sleep, diet, social networks, and physical activity—domains that are prioritized by older adults,^19–21^ which are identified in the literature as being at risk in older adults and as modifiable factors for dementia prevention.^20–22^ These domains were established as core components of the program, based on evidence of their significance, and were not subject to modification through user feedback. One of the program's goals was to educate participants on the relevance of these topics and their potential impact on brain health. The program successfully engages those interested in reducing the risk of dementia by addressing these relevant topics. The validation of BHPro's content is further underscored by participants’ feedback, which highlighted its perceived benefits for the broader community, referring to those seeking to enhance their brain health.

Focusing on prioritized domains that are highly relevant to older adults seeking to enhance brain health is essential; this approach allowed for more in-depth content on these topics, ensuring the presentation of information is both engaging and captivating for users. Effective delivery is crucial as it motivates individuals to delve deeper into the concepts presented. The validity of BHPro's content delivery and informational quality is corroborated by participant feedback, which indicate behavioral changes. For instance, participants reported modifications in their dietary habits, specifically reducing the consumption of high-fat and high-starch foods like French fries. Additionally, participants have placed greater emphasis on sustaining robust social relationships, further illustrating the program's impact.

### Mechanics

Mechanics, including user interface, design, visuals, and narration, played a critical role in the BHPro design. The program was co-designed with older adults, involving collaboration with CAG throughout the development.^17^ Additionally, a pre-pilot beta test group reviewed all functionalities, providing valuable feedback that informed improvements to the program's mechanics. The intuitive design and engaging features facilitated seamless navigation and user satisfaction, while clear visuals and effective narration enhanced comprehension and retention. Feedback from participants highlighted the importance of addressing varying language and technological proficiencies, with the program's responsive customer support team being a notable strength.

When developing an intervention focused on promoting brain health among older adults, it is imperative to prioritize mechanics—encompassing user interface, design, visuals, and narration—to ensure the tool's effectiveness and user engagement.^23^ An intuitive user interface is vital for facilitating seamless navigation, thereby reducing user frustration and enabling straightforward access to content.^23,24^ BHPro exemplifies excellence in website design through its attractive and appealing interface, which was seen to significantly enhance the user experience by making the platform more engaging and less intimidating. Engaging visuals, including images and videos, play a critical role in capturing attention and elucidating complex concepts, which can improve comprehension and retention of information.^24^ Moreover, effective narration provides auditory explanations that complement written content, catering to diverse learning styles and reinforcing key information.^25^ Pilot participants of BHPro commended its narration, noting that the voiceover was well-paced and of high quality.

For older adults, thoughtful design is particularly critical due to common sensory challenges such as decreased visual acuity and hearing impairments.^25^ Additionally, varying levels of technological proficiency among this demographic must be considered.^26^ For instance, a few participants encountered technical issues during the pilot testing of BHPro, which were related to content personalization and a notes column. Nonetheless, these were promptly addressed by the program's technical support team. The quick resolution of technical problems by support staff was a notable positive aspect, emphasizing the importance of responsive customer service in addressing user concerns. Corrections made during the pilot study were necessary in improving the program for the larger study, as the goal of the pilot was to identify and resolve such issues in preparation for the full study, aligning with the iterative process of program improvement recommended for such trials.

While these issues were typically resolved quickly through technical support, reliance on external assistance may limit sustainability in real-world settings where such support is less immediate. It is important to note that digital literacy varies among older adults, and those with limited technology experience may encounter greater barriers than were observed in this pilot sample. This aligns with existing literature demonstrating that lower digital literacy and technological barriers can hinder sustained engagement with web-based dementia prevention and cognitive health interventions,^27^ as well as broader digital health research identifying usability limitations and variable user competencies as persistent challenges.^28^

Features such as clear text sizes and color contrasts were seen to enhance accessibility and user comfort, while clear and engaging visuals contributed to ongoing motivation and interest.^24,25^ As BHPro addressed these design considerations, it was perceived as a well-constructed online intervention.

### Engagement and Learning

This study also suggests that BHPro fostered motivation and behavioral changes among users, empowering them to take charge of their health. Participants reported meaningful improvements in their lifestyles, such as dietary modifications and enhanced social engagement, demonstrating the program's efficacy in promoting self-management and preventive health measures. Moving forward, incorporating greater personalization could further enhance the program's effectiveness. Overall, BHPro exemplifies a promising approach to online brain health interventions.

The incorporation of engagement and learning factors—such as motivation, behavioral changes, perceived benefits, and self-management is of paramount importance.^29^ Motivation warrants sustained user engagement, and for BHPro participants, it inspired them to consistently interact with the program and integrate the knowledge gained into their daily routines. Participants were made more aware of how to steer their future by setting health goals and prioritizing certain areas that were not thought of before. For instance, participants are more cognizant of how much sleep they receive, have connected with old friends, and have found themselves to be more social and willing to engage.

These behavioral changes, facilitated by active engagement with the intervention, highlight the perceived benefits of BHPro. Learned strategies that are translated into daily routines reinforce the program effectiveness and usability. Additionally, empowering users to take charge of their own health through the intervention fosters self-efficacy and a proactive approach to managing risk factors of dementia.^7,29^ The qualitative themes observed, such as improvements in self-efficacy and knowledge, are further supported by quantitative measures in our companion manuscript.^14^ These results are consistent with findings from the pilot study,^10,13,14^ where similar improvements were evaluated and provide a solid foundation for the intervention's effectiveness. By promoting autonomy and providing users with the tools and knowledge necessary for effective self-management, BHPro supports long-term behavioral changes and preventive health measures. Ultimately, a digital tool that accounts for factors such as self-efficacy, knowledge and literacy, and empowerment ensures a more impactful and sustainable approach to improving brain health and reducing dementia risk in older adults.

### Limitations

The study's methodological limitations include the potential bias in self-reported data and the limited generalizability due to the sample size. Participants’ feedback was based on their subjective experiences, which may not fully capture the breadth of user experiences with BHPro. Additionally, the focus on a relatively small sample may not represent the diversity of potential users. For example, the utility of closed captioning in the program was not discussed as the current sample did not include persons with significant hearing loss. Some feedback was received that the program is slightly long, which could be considered a limitation as it may affect user engagement and overall satisfaction.

Future studies should aim for a larger and more diverse sample to enhance the generalizability of the findings. As a pilot study, it only focused on a 3-month timeframe, and longer-term issues, both positive and negative, could emerge with the full-length program. Furthermore, the pilot was delivered in a condensed 12-week format, which enabled exposure to the full curriculum and provided timely feedback on usability and content but did not allow for direct evaluation of adherence and motivation across the intended 45-week timeline. Although participants expressed strong motivation to engage during the pilot, sustaining engagement for 45 weeks may be more difficult. Prior studies related to digital dementia-prevention and lifestyle interventions indicate attrition rates of 20–40% in long-term programs, underscoring the challenges of maintaining engagement in older adult populations.^30,31^ As such, findings regarding acceptability should be interpreted with caution. These issues will be more comprehensively addressed in the ongoing CAN-THUMBS UP trial, in which BHPro is delivered in its full 45-week format.

Conducting focus groups over Zoom presented several challenges. Technical issues such as connectivity problems and difficulties in moderating discussions online may have affected the quality of the data collected. Non-verbal cues, which are often important in qualitative research, were less visible in a virtual setting, potentially impacting the depth of the insights gained. Future research could benefit from exploring alternative or supplementary data collection methods to mitigate these challenges.

### Next steps and program development

To build on the current findings, future research would do well to focus on refining personalization features and the incorporation of a glossary of terms.

The current study provides preliminary evidence of BHPro's favorable usability and perceived benefits as an online intervention for promoting brain health and reducing dementia risk among older adults. As digital solutions become increasingly integral to health and education, BHPro offers promising avenues for reaching and engaging individuals who are spending more time at home. The study highlights the importance of well-crafted content, effective design, and engaging delivery in ensuring the success of online educational tools. BHPro's content was positively received by participants, who appreciated its relevance, accessibility, and practical applicability to their daily lives. Addressing technical and personalization issues, as well as the challenge of adapting the program for individuals with varying levels of literacy identified will be crucial for the program's future development and success. Future iterations of BHPro should prioritize user-centered design enhancements, clearer troubleshooting resources, and integration of ongoing technical support to ensure accessibility and sustained engagement over the program's full 45-week delivery. By focusing on these areas, BHPro can better meet the needs of its users and contribute to improved health outcomes.

Lastly, beyond usability testing, the next phase of evaluation should assess the efficacy of BHPro in promoting lifestyle changes and reducing dementia risk, as well as examine outcomes such as cognitive function, adherence to recommended lifestyle behaviors, and improvements in brain health-related domains. These outcomes will provide critical evidence regarding the effectiveness of BHPro as a dementia-prevention and risk reduction intervention.

## Conclusion

This study provides preliminary evidence supporting the usability, feasibility, and perceived benefits of BHPro as a digital intervention to support dementia risk reduction among older adults. Participants valued the topics and accessibility of the content, the quality of design features, and the program's potential to promote motivation and behavior change. However, important considerations emerged, including the need for enhanced personalization, attention to digital literacy barriers, and strategies to sustain engagement over the full 45-week delivery. The accelerated 12-week pilot format allowed for comprehensive feedback but did not capture long-term adherence challenges, highlighting the importance of ongoing evaluation. These findings emphasize the significance of co-designing scalable, user-centered digital tools that can address modifiable dementia risk factors in aging populations. Future research, particularly through the CAN-THUMBS UP trial, should examine BHPro's efficacy in promoting lifestyle change, sustaining adherence, and ultimately contributing to the prevention of cognitive decline and improved brain health.

## Supplemental Material

sj-docx-1-dhj-10.1177_20552076251395585 - Supplemental material for Exploring the usability and perceived benefits of Brain Health PRO: An online educational program for healthy brain aging Supplemental material, sj-docx-1-dhj-10.1177_20552076251395585 for Exploring the usability and perceived benefits of Brain Health PRO: An online educational program for healthy brain aging by George Philip, Alexandra J Fiocco, Sylvie Belleville, Howard Chertkow, Howard H Feldman, Manuel Montero-Odasso, Haakon B Nygaard, Marie Y Savundranayagam and in DIGITAL HEALTH

sj-pdf-2-dhj-10.1177_20552076251395585 - Supplemental material for Exploring the usability and perceived benefits of Brain Health PRO: An online educational program for healthy brain aging Supplemental material, sj-pdf-2-dhj-10.1177_20552076251395585 for Exploring the usability and perceived benefits of Brain Health PRO: An online educational program for healthy brain aging by George Philip, Alexandra J Fiocco, Sylvie Belleville, Howard Chertkow, Howard H Feldman, Manuel Montero-Odasso, Haakon B Nygaard, Marie Y Savundranayagam and in DIGITAL HEALTH

## References

[1] Bacsu J Mateen FJ Johnson S , et al. Improving dementia care among family physicians: from stigma to evidence-informed knowledge. Can Geriatr J 2020; 23: 40. 10.5770/cgj.23.426 PMC7704071 33282053

[2] Alzheimer Society of Canada. Navigating the path forward for dementia in Canada. 2023. https://alzheimer.ca/sites/default/files/documents/Landmark-Study-Report-1-Path_Alzheimer-Society-Canada_0.pdf

[3] Fymat AL . Dementia: a review. J Clin Psychiatr Neurosci 2018; 1: 27–34.

[4] Paul C Mcdermott L Boehme G , et al. Aging people, aging places: Experiences, opportunities, and challenges of growing older in Canada. 2021.

[5] Livingston G Huntley J Liu KY , et al. Dementia prevention, intervention, and care: 2024 report of the Lancet standing Commission. Lancet 2024; 404: 572–628. 39096926 10.1016/S0140-6736(24)01296-0

[6] Kelly JT Campbell KL Gong E , et al. The internet of things: impact and implications for health care delivery. J Med Internet Res 2020; 22: e20135. 10.2196/20135 PMC7685921 33170132

[7] Fitzsimmons S Buettner LL . Health promotion for the mind, body, and spirit: a college course for older adults with dementia. Am J Alzheimers Dis Other Demen 2003; 18: 282–290. 14569645 10.1177/153331750301800504 PMC10833924

[8] Astell AJ Bouranis N Hoey J , et al. Technology and dementia: the future is now. Dement Geriatr Cogn Disord 2019; 47: 131–139. 31247624 10.1159/000497800 PMC6643496

[9] Anstey KJ Bahar-Fuchs A Herath P , et al. Body brain life: a randomized controlled trial of an online dementia risk reduction intervention in middle-aged adults at risk of Alzheimer's disease. Alzheimers Dement (N Y) 2015; 1: 72–80. 29854927 10.1016/j.trci.2015.04.003 PMC5974937

[10] Feldman HH Belleville S Nygaard HB , et al. Canadian Consortium on Neurodegeneration in Aging (CCNA), CAN-THUMBS UP Study Group. Protocol for the brain health support program study of the Canadian Therapeutic Platform Trial for Multidomain Interventions to Prevent Dementia (CAN-THUMBS UP): a prospective 12-month intervention study. J Prev Alzheimers Dis 2023; 10: 875–885. 37874110 10.14283/jpad.2023.65 PMC10258470

[11] Ostbye T Crosse E . Net economic costs of dementia in Canada. CMAJ 1994; 151: 1457. 7954140 PMC1337410

[12] Clemens J Parvani S . Canada must prepare for our aging population. COI:20.500.12592/1rqp30. 2017.

[13] Belleville S Chertkow H Feldman HH , et al. CCNA-CAN-THUMBS UP Study Group. Brain health PRO: an interactive, online, educational program for dementia. Alzheimers Dement 2023; 19: e075156.

[14] Belleville S Anderson ND Bherer L , et al. Brain health PRO/Santé cerveau PRO: the development of a web-based program for dementia literacy and risk factor reduction. J Prev Alzheimers Dis 2025; 12: 100134. 40090784 10.1016/j.tjpad.2025.100134 PMC12434268

[15] Herrman A . Focus groups. In: Allen M (ed.) The sage encyclopedia of communication research methods. Thousand Oaks, CA: Sage Publications, 2017, pp.579–581.

[16] Krueger RA . Focus groups: a practical guide for applied research. Thousand Oaks, CA: Sage Publications, 2014.

[17] D’Amico D Savundranayagam MY Biles R , et al. Engaging older adults in the process of aging research: a multimethod study evaluating the experience and efficacy of a Citizen Advisory Group for a dementia risk reduction program. Res Involv Engagem 2024; 10: 35. 39731188 10.1186/s40900-024-00643-6 PMC11674478

[18] Hubers C Lyons G . New technologies for the old: potential implications of living in later life for travel demand. Transp Policy (Oxf) 2013; 30: 220–228.

[19] Bennett GG Glasgow RE . The delivery of public health interventions via the Internet: actualizing their potential. Annu Rev Public Health 2009; 30: 273–292. 19296777 10.1146/annurev.publhealth.031308.100235

[20] Brito LMS Lima VAD Mascarenhas LP , et al. Physical activity, eating habits and sleep during social isolation: from young adult to elderly. Rev Bras Med Esporte 2021; 27: 21–25.

[21] Paterson DH Jones GR Rice CL . Ageing and physical activity: evidence to develop exercise recommendations for older adults. Appl Physiol Nutr Metab 2007; 32: S69–S108. 18213941

[22] Vodovotz Y Barnard N Hu FB , et al. Prioritized research for the prevention, treatment, and reversal of chronic disease: recommendations from the lifestyle medicine research summit. Front Med (Lausanne) 2020; 7: 585744. 33415115 10.3389/fmed.2020.585744 PMC7783318

[23] Volery T Lord D . Critical success factors in online education. Int J Educ Manage 2000; 14: 216–223.

[24] Bader JD Lowenthal PR . Using visual design to improve the online learning experience: a synthesis of research on aesthetics. In: Learner experience and usability in online education. Hershey, PA: IGI Global, 2018, pp.1–35.

[25] Piper AM Brewer R Cornejo R . Technology learning and use among older adults with late-life vision impairments. Univers Access Inf Soc 2017; 16: 699–711.

[26] Zhang S Grenhart WC McLaughlin AC , et al. Predicting computer proficiency in older adults. Comput Human Behav 2017; 67: 106–112.

[27] Tsai YIP Beh J Ganderton C , et al. Digital interventions for healthy ageing and cognitive health in older adults: a systematic review of mixed method studies and meta-analysis. BMC Geriatr 2024; 24: 217. 38438870 10.1186/s12877-023-04617-3 PMC10910826

[28] Conway A Ryan A Harkin D , et al. A review of the factors influencing adoption of digital health applications for people living with dementia. Dig Health 2023; 9: 20552076231162985. 10.1177/20552076231162985 PMC10017937 36937696

[29] Hartnett M . The importance of motivation in online learning. 2016.

[30] Atefi GL Koh WQ Kohl G , et al. Adherence to online interventions for family caregivers of people with dementia: a meta-analysis and systematic review. Am J Geriatr Psychiatry 2024; 32: 1271–1291. 38735829 10.1016/j.jagp.2024.04.008

[31] Soldevila-Domenech N Ayala-Garcia A Barbera M , et al. Adherence and intensity in multimodal lifestyle-based interventions for cognitive decline prevention: state-of-the-art and future directions. Alzheimers Res Ther 2025; 17: 61. 40098201 10.1186/s13195-025-01691-0 PMC11912746

