# Supplementary material for: Exploring the usability and perceived benefits of Brain Health PRO: An online educational program for healthy brain aging
Source: Digit Health. 2025 Nov 19;11:20552076251395585. doi: 10.1177/20552076251395585 (PMC12638707; doi:10.1177/20552076251395585)
Supplement: sj-docx-1-dhj-10.1177_20552076251395585 - Supplemental material for Exploring the usability and perceived benefits of Brain Health PRO: An online educational program for healthy brain aging [file sj-docx-1-dhj-10.1177_20552076251395585.docx]

Supplemental Table 1. Extracted Quotes by Theme and Subtheme

| Themes | Subthemes | Codes | Quote (participant ID) |
| --- | --- | --- | --- |
| Content | Likeability & Accessibility | General Content | “My experience was excellent. I found it very very positive” (P1) |
|  |  |  | “the program itself is excellent, it was well paced” (P1) |
|  |  |  | “Yeah I thought the program was very well done very well put together executed nicely” (P2) |
|  |  |  | “overall I enjoyed it I looked forward to having the challenges” (P7) |
|  |  |  | “There were some topics that I felt were probably less useful than others and one thing that I think that I'm running into now that I would like to see covered is… so many different websites so many different sources of information, Facebook and Google and all that stuff and it becomes overwhelming after a while you just can't keep up with all of it so I felt it should be addressed as maybe one of your topics” (P2) |
|  |  |  | “I think you have an unlimited number of people who are looking for ways to curtail what they're observing within their own thinking processes decreasing and I think again the positivity of the program is wonderful and I think you would find huge buy-in from people who are looking for ways to improve the quality of their life and maintain their brain power” (P5) |
|  |  |  | “generally, I'm pretty impressed with the program” (P3) |
|  |  |  | “I enjoyed going every Monday to the chapters. There was a lot of information there and I thought it was very positive” (P4) |
|  |  |  | “It wasn't as if you were inundated with a lot of information it was just sort of glimpses of things so I found that kept my interest up for one thing” (P5) |
|  |  |  | “It was excellent I mean I would say I would score this program with a gold star I mean I find it very user friendly and I'm not a computer type, I found it enjoyable I looked forward to the next week” (P5) |
|  |  |  | “I found [BHPro] incredibly useful and helpful, and I think it's going to be great for the community” (P7) |
|  |  |  | “I generally certainly enjoyed the program” (P8) |
|  |  |  | “I found the website really easy to follow” (P8) |
|  |  | Repetitive Content | “The only thing, and it’s probably just me, I just thought it was kind of repetitive” (P2) |
|  |  |  | “I did think it was a little too repetitive I mean I know that's what you need to do is the same exercises over and over but after a bit I just got tired of some of it I guess” (P6) |
|  |  | Accessibility | “that really helps you understand the context” (P1) |
|  |  |  | “I really appreciate the number of examples that are used in the presentation because that really helps you understand the context” (P1) |
|  |  |  | “it seemed doable and it seemed understandable and clear so no I felt comfortable with it, I mean some were challenging but that was the whole point of it!” (P2) |
|  |  |  | “I found [BHP] no problem. I thought it was very clear and easy to follow through and no problems, I didn't have any problem with the sound either so I think that was good” (P2) |
|  |  |  | “Well, I hate computers…your website was very easy to do, the weekly Monday lessons very good no problem, and the surveys on the cell phone were terrific as well so actually all that worked out very nicely” (P2) |
|  |  |  | “I agree as well. I found the website very easy” (P4) |
|  |  |  | “I find it very clear very understandable which is of course very important and something that I’ll repeat that was mentioned earlier, while I was taking this I was thinking of the challenge of people in their 20s and 30s and 40s being willing to accept this information. You know when I was in my twenties 30s and 40s, Dementia was just something that I heard of it that was out there, I guess I hoped I'd hit 70 one day and now here I am and I wish I had looked after my diet and exercise earlier than I did, luckily I'm OK, but the challenge is to get younger people to follow this regime to protect themselves”  (P6) |
|  |  |  | “I really particularly enjoy the mix of sort, of the medical information along with the recommendations. I really appreciate the way the recommendations are presented, they're very simply stated but very direct” (P6) |
|  |  |  | “was easy to get to and easy to understand the instructions and so forth” (P7) |
|  |  |  | “I had no issues with it. I kind of enjoyed it. I found it informative and the little challenges I think that goes along with it” (P3) |
|  |  | Accessibility – Language | “I would assume seeing as we’re such a diverse country, it will be available in multi languages, is that the intent? …Yeah see in this part of the world we've got a very high Chinese Asian population as well as East Indian, and Alzheimer's does not differentiate between race right. We all are subject to it therefore, cause I think that this would be an ideal program for all the health clinics throughout the country” (P1) |
|  |  |  | “I think some people would find the vocabulary quite difficult to comprehend if they were working alone at home, so that was just a caution” (P1) |
|  |  |  | “There might be some questions with vocabulary in some cases that people are not sure what cognitive impairment means or you know those kind of terms, even dementia you know maybe they need to be defined” (P2) |
|  |  |  | “I live in a rural community with many people here who have not a very extensive education, the language would detract from their willingness or wanting to be part of the program” (P5) |
|  |  |  | “the language would have to be modified because some of the language is fairly advanced I think. I live in a rural community with many people here who have not a very extensive education, the language would detract from their willingness or wanting to be part of the program” (P5) |
|  |  |  | “maybe a glossary of terms would be helpful” (P6) |
|  |  |  | “if English was my second or third language it may be an issue interpreting what is being said, you know so theoretically I suppose it would be best if this program was available in multiple languages…. no I found that there were minimal if any barriers to the program so I thought that was very well done” (P7) |
|  |  | Accessibility -Technology | “most of us have phones so that wasn't a big issue for me. The time was not onerous it didn't interfere with other things we had going on in our life we could do them when they were, so no I didn't find barriers to it” (P1) |
|  |  |  | “I'm sure there are a number of people, especially older people, who don't have that familiarity with cell phones that they assume that you have in today's world, so it's something to keep in mind that it may not be comfortable for a lot of people, or some people I should say not a lot, not this world” (P1) |
|  |  |  | “Well I loathe computers I'm terrified of them… I have found the weekly quizzes or whatever very easy to do on the computer, so the way it's set up to the program I think is very good even for people like myself who would prefer to write by hand than use a computer”. (P5) |
|  |  |  | “well for somebody that doesn't have a cell phone there's a learning curve that goes with it right” (P7) |
|  | Informative Nature of the Program | Informative Content | “I found the information good and as someone else mentioned, it wasn't Google type information it was relevant, it was supported it, was from articles that were peer reviewed, so yes I had a lot of respect for the creators of this program” (P1) |
|  |  |  | “It's very positive and it also gives you some solutions like you know doing the list of things write them down do things that help you remember lower your cholesterol level, all of those things are important and they're all doable so I can’t speak highly enough of how well I think this would lend itself to community” (P1) |
|  |  |  | “In general I think the topics you touched on were important and broad enough that it covered most of these things that we needed to know or wanted to know” (P2) |
|  |  |  | “I found the information very informative. I learned a lot about it” (P3) |
|  |  |  | “I really found a wealth of knowledge with this program so I thank you for that” (P6) |
|  |  |  | “There was a lot of information there and I thought it was very positive. Focusing on diet is very key for one, it's one of my interests” (P4) |
|  |  |  | “I found [BHPro] very educational, and I know that I've learned things that I'm going to continue to use…” (P5) |
|  |  |  | “The information was very good though and very informative and very helpful so I think it's a great program” (P6) |
|  |  |  | “I thought it was very informative. I liked the format of covering a number of different topics simultaneously” (P7) |
|  |  |  | “it is very very informative and I think people that have family members or friends that are going through this stage of dementia, this will really be an asset to log on and go through it” (P3) |
|  |  |  | “I thought the information was broad enough to cover a lot of different topics and a lot of people depending on where they are in life and so I thought it was very good” (P7) |
| Mechanics | Design | Visual-Graphics | “I think the graphics are great, the colour is fresh, it's uplifting, the photography is great, the font is easy to read. From a design standpoint I find it very appealing”(P3) |
|  |  |  | “I think it was attractive and bright” (P5) |
|  |  |  | “doing the coursework and the way it's presented I really had no issue with it at all and really quite enjoy them” (P6) |
|  |  |  | “there is an image of a jar of coins on its side with the coins spilled out and I noticed that they were American coins … and you know what I think you should change that photograph this is not any kind of rant or anything but it’s Canada they should be Canadian coins” (P6) |
|  |  | Narrator | “You couldn’t have had a better narrator” (P4) |
|  |  |  | “I really felt he was excellent…. very very good and his allocution was good and I found it calming, yes calming, but it was nice that he just had such a level cadence the whole time that it was very easy to follow him” (P5) |
|  |  |  | “I think his voice was very good because it was very well modulated, he spoke slowly I had no issues whatsoever, but I do have hearing aids” (P5) |
|  |  |  | “the narration was great, I never had no issues” (P8) |
|  |  | Reminder Texts and Emails | “I do appreciate that when I click on the link on the email it takes me to the website and then it takes me immediately into this week’s questions or lessons” (P1) |
|  |  |  | “it was helpful the one day … I totally forgot about doing it, that was the one time, and I heard the noise a couple of times and I thought, oh I didn't do this” (P4) |
|  |  | Notebook | “one time it said take notes or write a note and I would do that by hand I hadn't even realized there was a left column for notes so then the next time I did use the left column but I found going back and forth between the two pages was sort of frustrating so I then reverted back to doing notes by hand and sending” (P1) |
|  |  |  | “I didn’t find that column to put your notes in very useful either and didn’t use it at all in the end. It didn’t seem to me that it would enhance anything I was getting out of the program” (P2) |
|  |  |  | “I think as a user I would find it helpful for somebody to say, well what we've introduced this week is notebook and this is what you're supposed to do with it, as opposed to just finding it there” (P3). |
|  |  |  | “The only thing I would have changed was the notes. I think if it were populated with the questions that get asked in the components then you don't have to scramble and write down the question and then go back and make some notes” (P5) |
|  |  |  | “I noticed that the side panel where you list favorites or comments or whatever, I wasn't using at all and I sort of found it distracting to either make notes to enter later or to stop what I was doing and put my comments in there, so I didn't take advantage of that and I'm not sure if that's useful. I mean I do tend to use just pen and paper notes” (P7) |
|  |  | Sidebar Information | “I like the option of having the information there if we want to go back” (P1) |
|  |  |  | “I never found any issue and you could always go back and have a look which was a great set up on that program that if anybody wanted to review anything they could go back in and do it” (P3) |
|  |  |  | “I don't see the need to go to the other areas of it different chapters, just what relates to me that day or pertains to me that day” (P4) |
|  |  |  | “I only refer to that sidebar if there's a need to I guess and I haven't found much of a need to” (P6) |
|  |  |  | “I thought this is going to be good for some people that don't grasp things the first time I don't think you should do away with it, I think it'll be a tool for some people that may have the started Dementia that they can go back and refer to stuff” (P8) |
|  |  | Risk Profile - Speedometer | “it was really good to get the score for where we were assessed as green or yellow because that was really the only feedback we got on how we scored, and it was very relieving to see that I actually scored better than I had hoped that I would on those areas so yes that was very positive” (P1) |
|  |  |  | "the speedometer showing you, you know red yellow or green sort of thing, was a quick synopsis, so this is where I'm at and where I have to work on, so I thought the personalization was actually there right from the start" (P7) |
|  |  | Personalization | “... I think personally I feel that the personalization end of it could be improved to be of more value.” (P1) |
|  |  |  | “it's interesting that people are not picking up on the personalization because there is a place that you can go where it says based on the way that you've answered questions these are the things that we have identified as being areas of concern for you, … and then on the home page there were times when it would come up and say these are focus areas …which I think was what the subject matter was for the week but not necessarily for me” (P5) |
|  |  |  | “it would be useful to have a pin pointed to me to say more specifically, you know you're not as physically active as you should be and here are some suggestions” and “what foods am I missing, what sorts of activities should I be increasing” (P7) |
|  |  |  | “I felt the whole program was really personalized towards me because I had that input and I focused really on those three areas, the other areas I still felt important, so as far as personalization I think that’s really all I have to say. I felt I was being addressed as an individual rather than just this border plate same thing to everybody which I felt was positive” (P8) |
|  |  |  | “I was looking forward to actually a longer session you know like six months, but the sequencing and the timing and all that stuff could be adjusted slightly” (P7) |
|  |  |  | “I think it would be helpful to be able to print as you go through, there were times when there was something on the screen that I thought I'd like to keep that but there wasn't the option to be able to do that and I think that might be helpful for some people” (P5) |
|  |  |  | [personalization to access time] “7:00 o'clock in the morning which is early I guess but I'm up much earlier that I wouldn't mind being able to do it earlier, so I don't know if that's a possibility” (P2) |
|  |  |  | “offer for people to choose their time slot because I was away for three weeks during this last bit, I never travel with a computer but I had to take a computer with me to do the program, that wasn't terribly onerous but it just felt like if I had picked the time to do it I would not have picked a period when I was going to be away for three weeks, I would have picked a different time” (P1) |
|  |  |  | [length of program] “I enjoyed it throughout, but if it had gone on twice as long I think I would have found it a bit tedious”(P2)  *vs.*  “I think the period of time we had was right, maybe it could go a little longer but not too much longer” (P3) *vs.*  “I agree with the sentiment that it seems very very long. I was wondering while we were doing it if maybe one month segments of topic by topic may not be a better way to go” (P8) |
|  |  |  | “rather than just a 16 week program for people that do sign up if a monthly reminder every month as part of the program just to keep people in tune because it's human nature we all do it during the 16 weeks but then it kind of falls by the wayside we do need to be reminded” (P6) |
|  | Technical Issues | Glitches | “I would like to just throw in and maybe it was just my problem but when I was doing the tracking things, sometimes it wouldn't register when I hit the button that I meant to hit, and so I'd have to tap again or something which would slow me down and I found that quite frustrating” (P2) |
|  |  |  | “I didn’t find a lot of barriers. I found some glitches that were technical in nature and we worked through those and they were resolved, but they are the only barriers that I see” (P3) |
|  |  |  | “the technical things. I had to reload the website a couple of times. I uninstalled it reinstalled it” (P3) |
|  |  |  | “I agree the IT was very good I had some glitches and they were very good at fixing and taking care of it” (P6) |
|  |  | Tech support | “making sure especially at the beginning of the program that you have the human support, the support you need when you run into an issue … That initial support and continuing through is really really important for both your mental emotional approach to the program as well as your confidence level as well as your enduring through the program” (P1) |
|  |  |  | “[name of tech suppport] thank you for your help that first week because I couldn't get the whole screen to show up at all “(P5) |
|  |  |  | “I realized how important it is to have support for when glitches do occur and that it has to be quick and easy access to somebody so you can continue to move forward and not lose momentum so support is very important” (P7) |
|  |  |  | “Other than that sort of support which I really needed and really appreciated, it was prompt and made things happen, I don't know if this is going to be rolled out where there's maybe thousands of people taking it, I don't know what sort of help could be offered to if technical issues come up in that scenario, but as far as the support I had it was just outstanding” (P8) |
| Engagement and Learning | Motivation and Behaviour Changes | Motivation | "I found it fully engaging…I certainly am more aware of what I need to do for the future, it help set the path for where I'm going so I found it very helpful and I’m very glad to have participated...” (P7). |
|  |  |  | “I find it very positive very proactive very much geared towards saying to people you can make a difference, you need to put the effort into it but the effort will be rewarded, and I think that's what we all need to hear that it is good” (P5) |
|  |  |  | “There are little aspects of it that being proactive, to have this out at this time is really good” (P4) |
|  |  |  | “I woke up looking forward to it and looking forward to the evening one, I didn't miss any. So for the time I was involved I felt it was a nice challenge” (P2) |
|  |  |  | “I felt very engaged in the entire program. I felt that it was doing me a lot of good. A lot of what the program covers is things let’s say we have already have heard somewhere and know we should do but just the act of taking the program, I felt that I can either just listen to this again or I can actually change my habits and what I do, so it was very motivating for me that way. I’m a pretty good exerciser but I'm now exercising more” (P8) |
|  |  |  | “I suppose in a very mild sense it made me feel guilty about some of my behaviors and habits that may not be good for my brain *laughs*” (P7) |
|  |  |  | “honestly you get a much better study of your health completely health through the studies rather than just spending 10 minutes with your doctor” (P1) |
|  |  |  | “I really feel that everyone is responsible for maintaining their health to the best of their abilities and that hospital should be there for acute care, so I really am interested in learning how to best promote my own” (P5) |
|  |  |  | “Since I retired I've just lost touch with a lot of people and certainly my social circle has really reduced and it helped me recognize that that is probably not a healthy way to be, so the program really motivated me to go and reach out meet some new people” (P8) |
|  |  |  | “I'm getting older I'm retired now and I don't have as many social contacts, with COVID and so forth we are even more limited so you know it's been a worry for me and I thought that anything I can do to keep myself active and engaged is useful” (P7) |
|  |  |  | “I have this need to be active, to keep doing things” (P6) |
|  |  |  | “I'm getting older so I want to try to avoid it [dementia] as much as possible” (P2) |
|  |  |  | “I certainly am more aware of what I need to do for the future, it help set the path for where I'm going so I found it very helpful and I’m very glad to have participated thank you” (P7) |
|  |  |  | “I think the social component is incredibly important to this program because my own experience is that overtime especially since I retired, my social circle continues to shrink until there is hardly anybody left” (P1) |
|  |  |  | “it was a real feel good thing for me just to get confirmation that there is something that I can do to perhaps lessen the chances of contracting and coming down with Alzheimer's or at least delaying it or maybe reducing the severity” (P8) |
|  |  |  | “you gotta seek out other alternatives and keep that expansion there and that interest and that'll minimize so I've learned a lot from it and it’s going to be a pretty fruitful thing” (P3) |
|  |  | Behaviour Change | “I find when I'm out, I'm more chatty, and willing to engage other people. I’ve re-contacted some old chums and have got together with them so the engagement with that program did it for me.” (P8). |
|  |  |  | " One thing the brain help support program helped me do was it made me look at my food intake my health and what kind of food I was eating and so and so forth. I made a few adjustments and maybe will make a few more… Another thing it did that the program helped me do, I really look at how much sleep I really do get, so in the morning more so than I did before, so I know that I need that 6-8 hours sleep, and I usually do, I have always got that, but I make sure a little more.” (P3) |
|  |  |  | “since I've started this, I exercise more, I have definitely improved my diet, and it's been a reminder to me that you know, come on, you’ve slipped a little bit here, so that’s another huge benefit of the program” (P6) |
|  |  |  | “I've definitely made changes in my diet. We now have the Canada Food Guide up on the fridge, and I'm not eating the french fries that [spouse] is eating… The other big area for me has been activity. I have respiratory issues so a lot of exercise doesn't work for me but there were some very good clues on things that I could be doing at home that were low impact or less active kind of exercise so I found that very useful and I'm using that” (P5) |
|  |  |  | “I always ate pretty good but now I'm eating even better. I've already said that I'm really taking pains to be more engaged with people and again expand my social network, so the program has really affected my lifestyle that way diet and probably exercise as well” (P8) |
|  |  |  | “Well yes, the short answer is yes but I certainly wouldn't win any prizes for the degree of changes as far as diet, but it makes you more aware I suppose and in that sense there's more likelihood that you will in fact modify your behavior even a little bit to try and improve” (P1) |
|  | Perceived Benefits and Continuous Feedback | Perceived Personal Benefit | “I've learned a lot about minimizing some of the causes of dementia and keeping a whole bunch of frameworks open and it enhanced me to even research more online with it as well” (P3) |
|  |  |  | “I found it very educational and I know that I've learned things that I'm going to continue to use and I guess that's the point right” (P5) |
|  |  |  | “I found it very beneficial when I was doing it” (P8) |
|  |  |  | “I found it interesting that I’ve shared a lot of the information with [spouse] who is 10 years older than I am and it's been kind of an interesting happy hour conversation quite frequently, just some of the changes that we could be making as a couple, so that has definitely had an impact” (P5) |
|  |  |  | “I am going to take away from this certain things that I'm going to do for myself” (P3) |
|  |  |  | “I found out I learned a lot on some of the subjects …[when speaking to spouse] I said ‘oh did you know about this, or that’ so I must have been learning a lot from what the topics that you were covering. So I found it very very helpful for my myself”  (P8) |
|  |  |  | “it does stimulate conversations about brain health in general, not just dementia and Alzheimer’s but just overall brain health and the challenges they face and the coping mechanisms and so it did stimulate larger conversations about brain health in general and I thought that was very good” (P7) |
|  |  |  | [no perceived benefit] “Has it impacted my daily life? Probably not at all. Was I very glad to do it? 100%... I guess it's reassured me” (P1) |
|  |  | Perceived Benefit for Others | “I think it should be spread out and used more, and I think it would be very beneficial to a lot of people and I also think you should try to catch people early, like maybe even in their 50s or something” (P2) |
|  |  |  | “I agree that this would be an excellent program for rolling out to the community in time” (P7) |
|  |  |  | “I agree that there was a lot of interest from the community and my fellow friends and stuff because most of us are at that age where this is a concern whether you consciously think about it or not… so any opportunities to try and prevent help reduce all of these things I think is just going to be so welcoming for the communities” (P7) |
|  |  | Continuous Feedback | “I was actually thinking that it would be really great if they did a yearly thing so that we could compare our performance, see whether there's a deterioration or not, and I think that program could be adapted to being more of a score, I mean sort of not necessarily all the educational components but more of the tests and the quizzes” (P1) |
|  |  |  | “I'd like to have some feedback and the comparison idea is a very good one overtime, and maybe more elaboration on some of the low points and high points” (P2) |
|  |  |  | “I do think a year from now I would like to know where I am and given a chart or given a range would make me feel very comfortable” (P4) |
|  |  |  | “just like a six month or 12 month follow up as well just to check in to see whether I've actually worked on the things that I needed to work on and it's making a difference, has there been any kind of deterioration, any alarm bells that I should be aware of, so definitely checking in” (P5) |
|  |  |  | “a one year check-up sort of thing would be good as to know how we're progressing or not as the case may be and I think I would enjoy that” (P6) |
|  |  |  | “I would definitely continue with this and would like to. I need a reminder I think most people need a reminder, we received this education let's say an exercise so we're motivated and we get out and do it but then you know we have snow in April and you miss a day and we need to be reminded that we need to do this so I would appreciate that, certainly being a part of a program where every month or every two months that you get a reminder email and if you want to take a segment again take it again, but I would certainly like to continue this into the future” (P8) |
|  |  |  | “If we’re doing follow ups which I think is a very good idea, I would like to see places identified where our scores are lower than expected or dropping and maybe highlighted, or even with suggestions on ‘this is something you actually need to practice a little more on and here are some more refined kinds of practice you can do’. That would be useful” (P2) |
|  |  |  | “I think it would be fair that they provide us with some kind of information as to how we did, I really do” (P4) |
|  |  |  | [referring to cognitive scores] “I would have liked to have a little feedback for example was I doing better in the mornings or evenings that kind of thing you know would have come out of the results it would have been interesting to me” (P2) |
|  |  |  | [referring to cognitive scores] “I'm someone who always likes to get results so I found that a little frustrating but it was upfront that we would not get results… So it's just because to me being proactive with your health is also dealing with the results you get in exams so that you can make improvements that are very pertinent to your situation, so that's the only thing I would say” (P1) |
|  |  |  | “One of the things that I found were the indicators of you need help with this you're doing well and whatever, you don't get any feedback what might- you know suggestions to improve your score in that particular category that would be really useful, you know what foods am I missing what sorts of activities should I be increasing … It could be but it would be useful to have a pin pointed to me to say more specifically, you know you're not as physically active as you should be and here are some suggestions”  (P7) |
